# The Growth Arrest-Specific Transcript 5 (GAS5) and Nuclear Receptor Subfamily 3 Group C Member 1 (NR3C1): Novel Markers Involved in Multiple Sclerosis

**DOI:** 10.22088/IJMCM.BUMS.7.2.102

**Published:** 2018-05-07

**Authors:** Jalal Gharesouran, Mohammad Taheri, Arezou Sayad, Soudeh Ghafouri-Fard, Mehrdokht Mazdeh, Mir Davood Omrani

**Affiliations:** 1 Department of Medical Genetics, Shahid Beheshti University of Medical Sciences, Tehran, Iran.; 2 Urogenital Stem Cell Research Center, Shahid Beheshti University of Medical Sciences, Tehran, Iran.; 3 Neurophysiology Research Center, Hamadan University of Medical Sciences, Hamadan, Iran.; & First two authors contributed equally in the writing of the paper.

**Keywords:** Multiple sclerosis, GAS5, NR3C1

## Abstract

Recent studies have revealed that long noncoding RNAs (lncRNAs) are connected with pathogenesis of neurodegenerative diseases. Additionally, glucocorticoids have fundamental regulatory roles on the immune system, and act as potent therapeutic compounds for autoimmune and inflammatory diseases. The long noncoding RNA growth arrest-specific 5 (*GAS5*) which accumulates inside the cells in response to cellular starvation/growth arrest, acts as a potent repressor of the glucocorticoid receptor (GR) through its glucocorticoid response element (GRE). The aim of the present study was to investigate the role of lncRNA *GAS5* and its downstream target Nuclear Receptor Subfamily 3 Group C Member 1(*NR3C1*) in the pathogenesis of multiple sclerosis (MS), and to define the role of *GAS5* in the regulation of *NR3C1* expression. Quantitative polymerase chain reaction was performed for investigating the expression of *GAS5* and *NR3C1* in MS patients and healthy subjects. We found that *GAS5* levels were up-regulated in the MS patients, blood compared with healthy subjects in correlation with *NR3C1* expression. Our findings suggest that* GAS5* may play on important role in the molecular etiology and treatment of MS.

Multiple sclerosis (MS) is a multifocal chronic progressive inflammatory demy-elinating disease of the central nervous system (CNS) that leads to severe neurological disability through axonal degeneration and gliosis (1). Recent studies have reported that human genome encodes many long non-coding RNAs (lncRNAs) (2). LncRNAs participate in multiple biological mechanisms and regulate gene expression via transcriptional, post-transcriptional, and epigenetic regulation. Most of lncRNAs have tissue-specific expression pattern with precise temporal and spatial regulation in the CNS. Dysregulation in lncRNAs expression has been shown to be associated with different types of neurodegenerative disorders such as MS, Alzheimer's, Parkinson's and Huntington's diseases. However, the exact mechanisms of such contribution have remained unclear.The growth arrest-specific 5 (*GAS5*), a small nucleolar RNA (snoRNA) host gene, is accumulated in cells showing growth arrest upon lack of nutrients or growth factors. This lncRNA has been detected for the first time through screening of potential tumor suppressor genes expressed at high levels during growth arrest (3). *GAS5* has multiple functions at cellular level such as cell cycle arrest control at the G0/G1 phase, alteration of protein synthesis, and modulation of apoptosis (4). This gene encodes 10 box C/D snoRNAs within 11 introns and has been considered as a member of the 5′-terminal oligopyrimidine tract (5′ TOP) gene family. The nutrient's levels alter cellular growth and survival through affecting gene transcription.

The transcription of *GAS5* is increased during growth arrest due to the presence of anti-translation inhibitors, and serum starvation. Such alteration in *GAS5* expression prepares cells for apoptosis via inhibition of glucocorticoid-mediated induction of numerous responsive genes. *GAS5* RNA, despite of little protein-coding potential, is spliced, polyadenylated, and associated with ribosomes.* GAS5* has been recently reported to act as a starvation- or growth arrest-linked riborepressor with a central role in the glucocorticoid receptor (GR) regulation. *GAS5* binds to the GR DNA-binding domain and interferes with glucocorticoid response element (GRE) (5, 6).

Glucocorticoids modulate gene transcription, and have roles on cell growth, energy expenditure, and cell survival as well. They are vastly used in the treatment of inflammatory and autoimmune diseases. Upon binding with GRs, they translocate into the nucleus, and participate in the transcriptional activity of target genes by binding to specific promoter DNA sequences. They have interaction with transcription factors, and different signaling pathways. In this current study, we examined the expression of *GAS5* in correlation with one of downstream targets called Nuclear Receptor Subfamily 3 Group C Member 1 (*NR3C1*) which encodes for a glucocorticoid receptor.

## Materials and methods

**Study design and eligibility criteria**

Blood samples were collected from 50 healthy volunteers (27 female and 23 male, mean age: 35.3 ± 2.1 years) and 50 relapsing remitting (RR) MS patients in early stage of the disease (31 female and 19 male, mean age: 36.2 ± 2.9 years, age of onset: 31.36 ± 2.4 years, duration of disease: 6.1 ± 3.1 years, expanded disability status scale EDSS: 2.72±2.3). Exclusion criteria were pregnancy, steroid therapy or infection within one month prior to sample collection, existence of other autoimmune diseases, malignancies or chronic infectious diseases. Inclusion criteria for RR-MS patients were diagnosis of RRMS according to the revised McDonald’s criteria, and stable phase of the disease (7). Exclusion criteria for RR-MS were clinical or radiological relapse within one month from sample collection. We also excluded those with smoking history. No significant difference was seen in body mass index between cases and healthy controls.

**Blood sampling**

Whole blood samples were collected from participants after acquiring informed consent form. Ethics approval for the study was acquired from the local ethics committee of Shahid Beheshti University of Medical Sciences (No. 3275). Blood samples were collected from MS patients in Iranian MS society clinic and Imam Hossein Hospital in Tehran. Total RNA was extracted using Geneall Hybrid-RTM blood RNA extraction Kit (cat No. 305-101) according to the manufacturer’s instructions. Extracted RNA was subjected to complementary DNA (cDNA) synthesis using Geneall cDNA synthesis kit according to the manufacturer’s instructions. Total RNA was treated with DNase I for 30 min at 42 °C. Allele ID 7 software (Premier Biosoft, PaloAlto, USA) was used to design the specific probes and primers. *HPRT1* as a housekeeping gene was used for normalization of the gene expression level of samples. The sequence of probes and primers are presented in Table 1.

**Quantitative real-time PCR (TaqMan®)**

Expression levels of *GAS5* and its target *NR3C1* were assessed by real-time RT-PCR TaqMan® analysis using the Corbett Rotor gene 6000 instrument (Corbett Life Science). The reverse transcription reaction was carried out with the high capacity RNA-to-cDNA kit (Geneall) and the real-time PCR was performed in duplicate using the TaqMan® gene expression assay according to the manufacturer’s instructions. The thermal cycling conditions for TaqMan assays were as follows: 10 min at 95 °C followed by 40 cycles at 95 °C for 15 s and 60 °C for 45 s. The expression levels of *GAS5 *and *NR3C1* were evaluated using the comparative Ct method (2^-ΔΔCt^ method). Ct values were corrected based on PCR efficiencies using LinReg PCR. The *GAS5* and *NR3C1* expression values were normalized using the housekeeping gene. Quality control was done using negative control in every reaction.

**Statistical analysis**

SPSS Version 18 (Chicago, IL, USA) statistical package was applied for statistical analysis. Independent t test was done to compare the differences between the results of two groups. Pearson correlation coefficient was used for the evaluation of the correlation between variables. P values less than 0.05 were regarded as significant. Spearman rank order correlation test was considered to evaluate the possible correlation between relative expression levels of genes and clinical variables as well as between the levels of gene expression in patients.

## Results

**Demographic data of participants**

Clinical characteristics, disease duration, and demographic characteristics of RRMS patients and healthy controls are presented in Table 2. Participants were classified into three different groups based on their age ranges (<30, 30–40, and>40 years), and sex (male or female). Separate computations were done for the total numbers in both groups. All patients were treated with interferon (IFN)-β for at least two years (intramuscular injection of 20 μg of CinnoVex three-times a week), and were recognized as IFN-β responders (9).

***GAS5*** **and** ***NR3C1*** **expression levels**

The expression level of *NR3C1 *has been compared between MS patients and control group based on age, and sex of the participants. Obtained results are presented in Table 3. Statistical analysis showed significant up-regulation of *NR3C1 *gene in MS patients compared with healthy controls (P=0.03), and in male subgroup compared with the corresponding age and sex-matched healthy subjects (P = 0.001).

**Table 1:** The sequence of probes and primers

**Gene name**	**Primer and probe length (bp)**	**Primer and probe sequences**	**Product length (bp)**
*HPRT1*	18	F: AGCCTAAGATGAGAGTTC	88
	21	R: CACAGAACTAGAACATTGATA	
	24	FAM -CATCTGGAGTCCTATTGACATCGC- TAMRA	
*GAS5*	20	F: CTGCTTGAAAGGGTCTTGCC	98
	21	R: GGAGGCTGAGGATCACTTGAG	
	24	FAM- ACCCAAGCTAGAGTGCAGTGGCCT- TAMRA	
*NR3C1*	22	F: AGAGGAGGAGCTACTGTGAAGG	78
	21	R: TCGCTGCTTGGAGTCTGATTG	
	24	FAM -TGCGTCTTCACCCTCACTGGCTGT- TAMRA	

**Table 2:** Demographic information of RRMS patients and healthy controls

**Variables**	**MS patients**	**Controls**
Female/male [no. (%)]	31(62%)/19(38%)	27(54%)/23(46%)
Age (mean ± SD, year)	36.2 ± 2.9	35.3 ± 2.1
Age at onset (mean ± SD, year)	31.41 ± 2.8	-
Duration (mean ± SD, Year)	4.58 ± 3.2	-
EDSS (mean ± SD)	3.07 ± 2.7	-
EDSS: expanded disability status scale.		

**Table 3:** *NR3C1 *expression levels in MS patients compared with control group, based on age and sex of the participants

***NR3C1*** ** expression**		**Controls no.**		**MS patients no.**		**Expression ratio**	**Standard error**	**P value**		**95% confidence interval**	
Total			50		50	1.9751	0.34		0.03		[0.44, 1.97]
Male			23		19	3.2544	0.404		0.001		[0.98, 3.05]
Female			27		31	1.3278	0.325		0.538		[-0.57, 1.31]
<30 years	Male		6		4	1.7151	0.295		0.476		[-2.17, 3.91]
	Female		3		7	2.3501	4.65		0.183		[-9.87, 12.5]
30–40 years	Male		8		5	5.5470	0.91		0.042		[0.253, 0.75]
	Female		5		10	1.5884	1.14		0.679		[-2.27, 3.85]
>40 years	Male		9		10	3.2386	-1.01		0.038		[0.027, 4.2]
	Female		19		14	1.0221	0.39		0.788		[-1.43, 1.27]

**Table 4:** *GAS5 *expression levels in MS patients compared with control group, based on age and sex of the participants

***GAS5*** ** expression**		**Controls no.**	**MS ** **patients no.**	**Expression ratio**	**Standard error**	**P value**	**95% confidence interval**
Total		50	50	0.6687	0.08	0.061	[-1.19, 0.08]
Male		23	19	0.8945	-0.227	0.561	[-1.25, 0.74]
Female		27	31	0.5459	0.411	0.061	[-1.84, 0.16]
<30 years	Male	6	4	0.9636	-0.01	0.476	[-4.16, 3.73]
	Female	3	7	0.1920	5.13	0.067	[-16.7, 10.3]
30–40 years	Male	8	5	1.7716	0.33	0.171	[-0.56, 2.77]
	Female	5	10	0.6850	1.11	0.206	[-3.05, 1.79]
>40 years	Male	9	10	0.5529	-0.96	0.156	[-2.93, 0.72]
	Female	19	14	0.5677	0.16	0.226	[-2.17, 0.74]

*GAS5 *mRNA level was not significantly different in MS patients compared with healthy subjects. The results of the *GAS5 *expression level in MS patients in comparison with control group are shown in Table 4.

**Correlation between** ***GAS5*** **and** ***NR3C1*** **expres-sion levels and clinical characteristics of patients**

*GAS5* gene expression level did not show significant correlations with EDSS, disease duration, or age at onset (Figures 1–4). Also, there were no significant correlations between *NR3C1 *expression level and disease duration, or age at onset (Figures 5-8).

**Correlation between expression levels of** ***GAS5*** **and** ***NR3C1*** **genes**

Our results have shown a significant correlation between *GAS5* and *NR3C1* expression levels (Fig.9).

## Discussion

Recent studies have shown that lncRNAs are involved in the development and progress of human diseases such as neurodegenerative diseases (10, 11). Certain circulating lncRNAs, such as inflammation regulators, and immune response, have been identified as useful biomarkers for neurodegenerative disorders as well as pathway prediction and determination (12, 13).

**Fig. 1:**
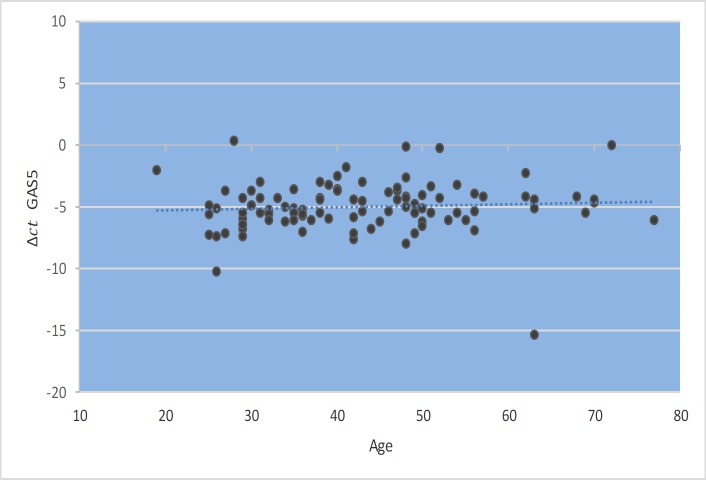
Spearman correlation between *GAS5 *expression and age.

**Fig. 2:**
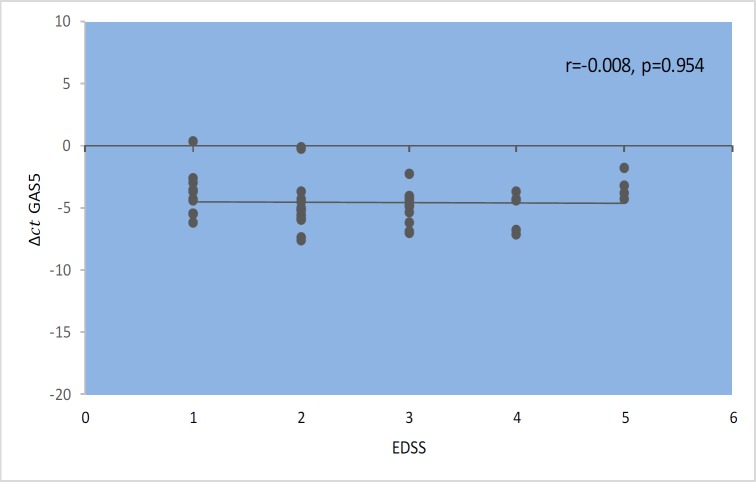
Spearman Correlation between *GAS5 *expression and EDSS in patients .

**Fig. 3:**
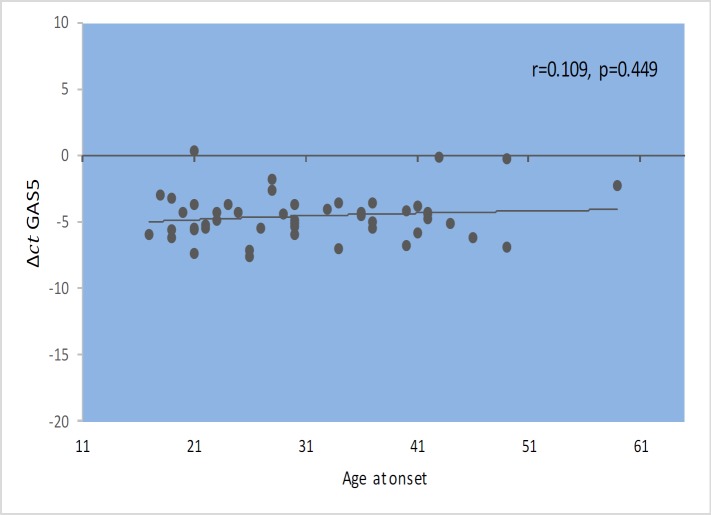
Spearman correlation between *GAS5 *expression and age at onset in patients.

**Fig. 4:**
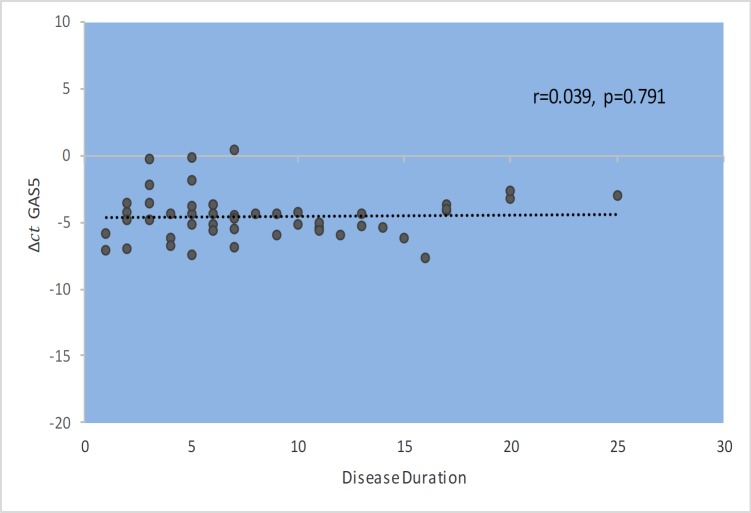
Spearman correlation between *GAS5 *expression and disease duration in patients.

**Fig. 5:**
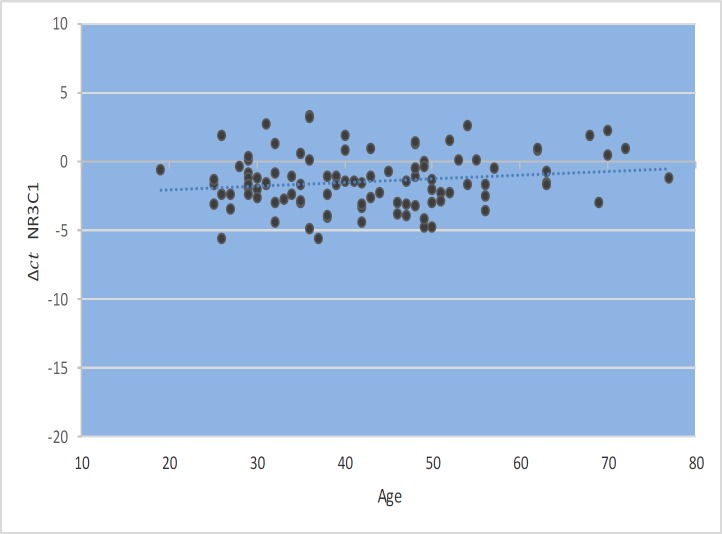
Spearman correlation between *NR3C1 *expression and age.

**Fig. 6:**
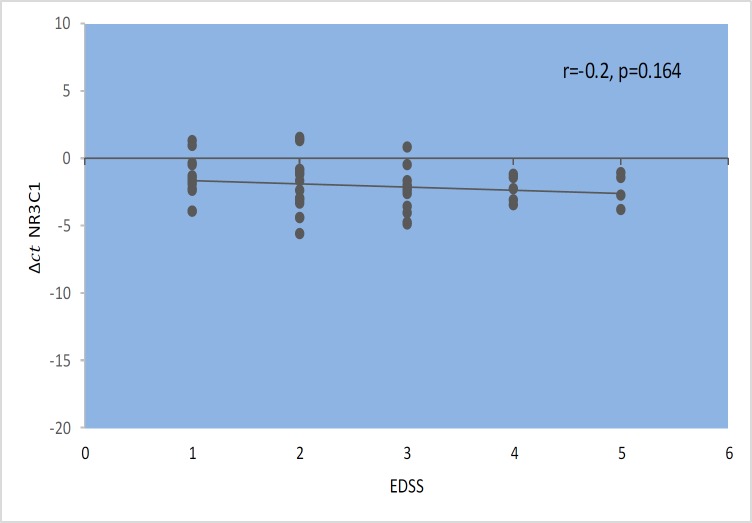
Spearman correlation between *NR3C1 *expression and EDSS in patients.

**Fig. 7:**
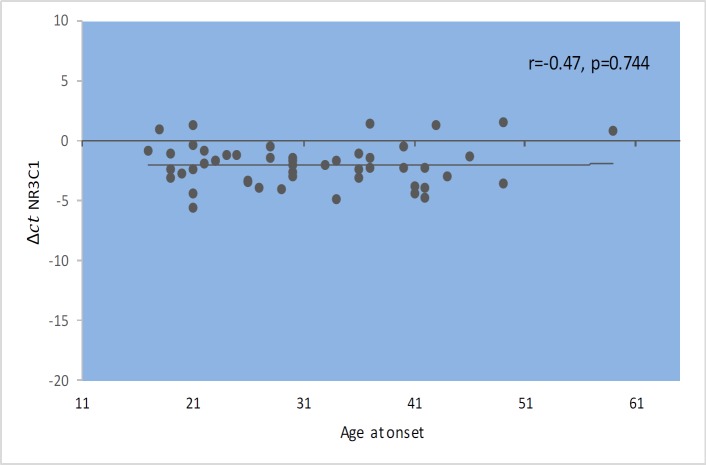
Spearman correlation between *NR3C1 *expression and age at onset in patients

**Fig. 8:**
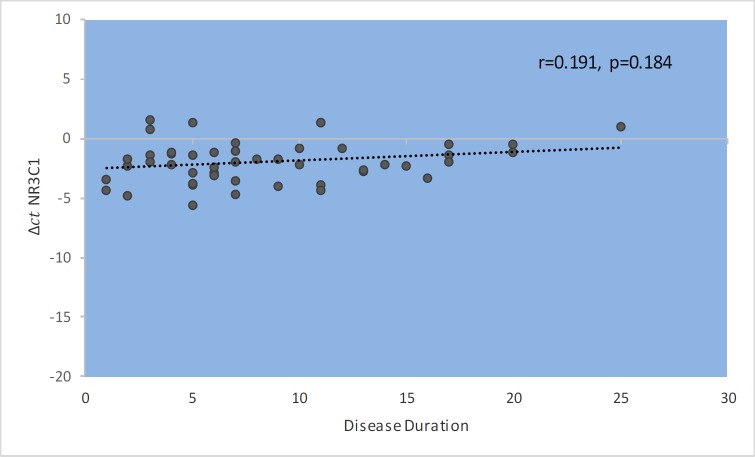
Spearman correlation between *NR3C1 *expression and disease duration in patients

**Fig. 9:**
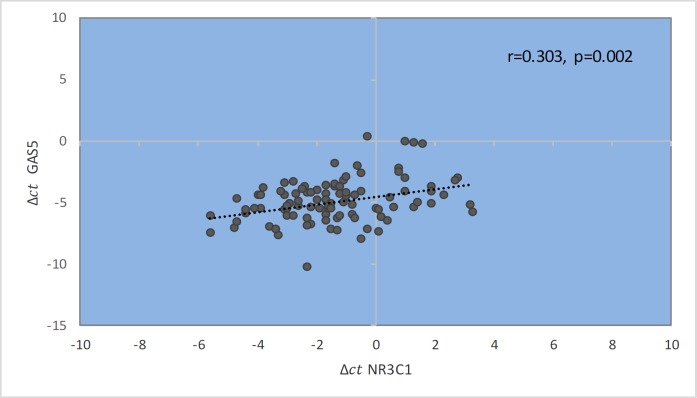
Spearman correlation between expression levels of *GAS5 *and *NR3C1*.

**Fig. 10:**
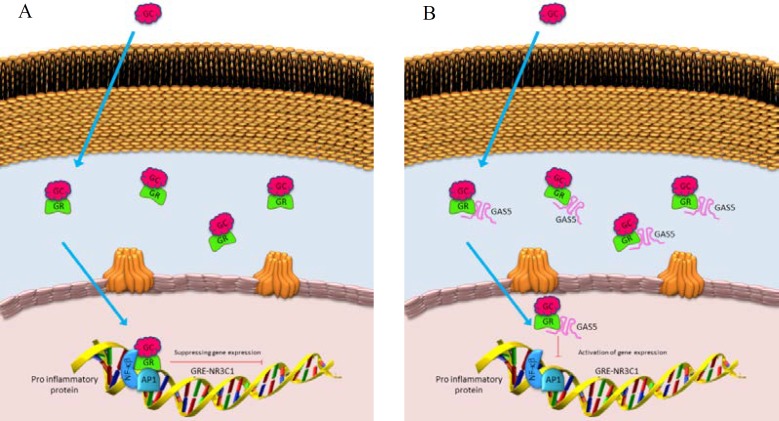
Mechanism of *GAS5 *(A) and *NRC3C1 *(B) contribution in proinflammatory pathway genes expression.

*GAS5* is a lncRNA involved in the regulation of cell cycle with central role in normal growth arrest in T-cell and non‑transformed lymphocytes (14). *GAS5* has a conserved 5'‑terminal oligopyrimidine tract (5'‑TOP) that may explain its cellular accumulation upon growth arrest in a range of species (15). Recent studies hawe reported the role of *GAS5* in many types of cancer (16, 17). Functional studies have shown that *GAS5* inhibition may suppress cell apoptosis, whereas overexpression increases cell apoptosis and reduces the rate of progression through the cell cycle. It hase been concluded that modulated* GAS5* is required for normal growth arrest (18).

On the other hand, glucocorticoids are commonly applied in inflammatory, autoimmune disorders, and in the prevention of rejection in transplanted patients (19, 20). Their efficacy is different between individuals, and the side effects have been shown in recent reports (21). Glucocorticoids regulate gene expression of target cells through binding with the *NR3C1 *encoded GR. The *NR3C1* gene has a C-terminal ligand-binding domain (LBD), an N-terminal transcriptional regulatory region, and a central DNA binding domain (DBD) (22). GR autoregulation by a negative feedback mechanism has been identified through the binding of the activated receptor to intragenic sequences called GRE-like elements contained in GR. Along with these findings; interaction of *GAS5* with the activated GR suppresses its transcriptional activity through preventing its association with GRE targets (6).

In the present study, correlation analyses between *GAS5* lncRNA expression levels and clinical data of MS patients revealed no significant correlation between its expression levels with age, age at onset, EDSS, and disease duration. Also, we found correlations between the expression levels of *NR3C1* and clinical data of RRMS patients. However, expression ratio in MS patients hase increased by 1.9 times, and this increase was significantly different between two groups (P=0.03). The results of our study show an increase in the *NR3C1* expression by about 3.2 times in affected males compared with the control group (P=0.001). Moreover, this upward trend increases the expression of the gene in a manner consistent with age. The peak in the range between 30 and 40 years in men is up to 5.5 fold, which is statistically significant (P =0.042). In addition, we found higher expression level of *GAS5* in male MS patients in the same age range compared with male controls, although the difference was not significant. Such finding might be related to higher threshold for initiation of MS in males. On the opposite side, the increase in the expression of this gene in women with a disease was lower than that of men.

It can be concluded that *GAS5* and its downstream target, *NR3C1*, might participate in a complex multigene interaction network which regulates the expression of several targets in the pathways that may be deregulated in MS. It has been recently reported that the nonsense-mediated degradation (NMD) pathway can regulate the function of *GAS5* in mammalian cells (22). Interestingly, the ability of *GAS5* in binding to GR will result in the inhibition of its ligand dependent association with DNA. Along with this action, GR binds the promoters of various glucocorticoid responsive genes, including inflammatory pathway genes (Figure 10 A and B). The mechanism of GR anti-inflammatory effects is through its interaction with other transcription factors such as activator protein 1 (AP1) or nuclear factor κB, thereby trans-repressing their transcriptional activity, which leads to inhibition of pro-inflammatory transcription factors. It has been shown that poor responders to glucicorticoids have higher levels of *GAS5* and *NR3C1* in comparison with good responders (23, 24). It seems that abnormal expression levels of *GAS5* may modify glococorticoid effectiveness by interfering with the mechanism of GR autoregulation. The present study provides a novel finding that *NR3C1* gene expression in the peripheral blood mononuclear cells is modulated by *GAS5*. We found a significant correlation between *GAS5* and *NR3C1* expression levels (figure 5). Our results showed that there is a direct and moderate relation between the expression of these two genes, so that by increasing the expression of each, the expression of the other one also increases, which is quite significant (r= 0.3, P = 0.002).

This clinical study prepared the foundation for decoding the molecular pathways, and genetic mechanisms underlying the pathology of MS disease. For this reason, assessing lncRNAs as important regulators of gene expression will help to identify the gene targets of *GAS5* in the context of MS. LncRNAs including *GAS5* are multifaceted and have complex functional responsibilities which are often dictated by the cell type and molecular targets. This is the first study that investigated and correlated levels of circulating lncRNA *GAS5* with downstream target *NR3C1* in MS. In conclusion, we proposed the altered expression of *GAS5 *as a pathologic event in MS which might lead to alteration of the *NR3C1* gene function or expression. The design may be easily adapted to larger series and in patients with other chronic inflammatory, and autoimmune diseases.* GAS5* can be considered as a candidate marker of glucocorticoid resistance in such disorders. In addition, large association studies are needed to assess the effects of *GAS5 *and *NR3C1* functional polymorphisms in conferring risk to MS. These results can be expanded to verify the model that by combining *GAS5* levels with other molecular markers such as NR3C1 levels may predict onset of MS.

## Conflict of interest

The authors declared no conflict of interest.
